# Targeting a Lipid Desaturation Enzyme, SCD1, Selectively Eliminates Colon Cancer Stem Cells through the Suppression of Wnt and NOTCH Signaling

**DOI:** 10.3390/cells10010106

**Published:** 2021-01-08

**Authors:** Yeongji Yu, Hyejin Kim, SeokGyeong Choi, JinSuh Yu, Joo Yeon Lee, Hani Lee, Sukjoon Yoon, Woo-Young Kim

**Affiliations:** 1 College of Pharmacy, Sookmyung Women’s University, Seoul 04312, Korea; djmd3353@naver.com (Y.Y.); hyejin9133@gmail.com (H.K.); choi9174@gmail.com (S.C.); jinsuhyu@sookmyung.ac.kr (J.Y.); jooyeon0602@naver.com (J.Y.L.); HLee23@mdanderson.org (H.L.); 2 Research Institute of Women’s Health, Sookmyung Women’s University, Seoul 04310, Korea; yoonsj@sookmyung.ac.kr; 3 Research Institute of Pharmaceutical Sciences, Sookmyung Women’s University, Seoul 04312, Korea

**Keywords:** CSC, SCD1, monounsaturated fatty acid, NOTCH, Wnt

## Abstract

The elimination of the cancer stem cell (CSC) population may be required to achieve better outcomes of cancer therapy. We evaluated stearoyl-CoA desaturase 1 (SCD1) as a novel target for CSC-selective elimination in colon cancer. CSCs expressed more SCD1 than bulk cultured cells (BCCs), and blocking SCD1 expression or function revealed an essential role for SCD1 in the survival of CSCs, but not BCCs. The CSC potential selectively decreased after treatment with the SCD1 inhibitor in vitro and in vivo. The CSC-selective suppression was mediated through the induction of apoptosis. The mechanism leading to selective CSC death was investigated by performing a quantitative RT-PCR analysis of 14 CSC-specific signaling and marker genes after 24 and 48 h of treatment with two concentrations of an inhibitor. The decrease in the expression of Notch1 and AXIN2 preceded changes in the expression of all other genes, at 24 h of treatment in a dose-dependent manner, followed by the downregulation of most Wnt- and NOTCH-signaling genes. Collectively, we showed that not only Wnt but also NOTCH signaling is a primary target of suppression by SCD1 inhibition in CSCs, suggesting the possibility of targeting SCD1 against colon cancer in clinical settings.

## 1. Introduction

Cancer is one of the leading causes of death in most countries. Metastasis is responsible for most of those cancer-related deaths because the spread tumors mostly do not respond to the currently available treatment [1,2,3]. A small population of the cells in malignant tumors called cancer stem cells (CSCs) have been identified as the origin of cancer evolution [4,5,6,7]. More importantly, numerous evidences from preclinical and clinical data support that the CSCs are responsible for tumor recurrence after conventional chemotherapy/radiation-therapy and metastasis [2,3,4,5,6]. When recurring or initiating new colony formation in secondary tissues, the CSCs may evolve in different and diverse direction, making it quite a different and difficult disease to cure [3,6,7].

Although many studies have focused on searching for a selective target for CSCs to achieve better outcomes of conventional chemotherapy, radiation therapy, and targeted therapy, only a few are currently under clinical evaluation [6]. Therefore, the identification of new vulnerable targets for CSCs and the development of therapeutic approaches may be urgently required.

Originally, the consumption of glucose by cancer cells was identified and described approximately 100 years ago [8]. More new findings on the altered metabolism of cancer cells have allowed researchers to utilize these changes to determine the diagnosis and treatment [9,10]. The metabolic reprogramming in CSCs is quite different from bulk cancer cells (BCCs), which are a mixed population including very small numbers of CSCs and a large proportion of differentiated cancer cells. According to recent evidence, CSC-specific targeting may be feasible by blocking some metabolic pathways that are only essential in CSCs [11,12,13,14,15]. In particular, the lipid anabolic pathways have recently attracted interest as a selective target for cancer research [16].

In a recent study, we and other groups showed a greater monounsaturated fatty acid (MUFA) composition in CSCs than in BCCs, suggesting a role for the MUFA-generating enzyme, stearoyl-CoA desaturase 1 (SCD1) in ovarian, brain, and colon CSCs [11,17,18]. We also presented the clinical and biochemical relevance of targeting SCD1 in colon cancer [11] based on analyses of TCGA data and mass spectrometry data, respectively. Several reports have described the efficacy of SCD1 in cancer cell proliferation and proposed mechanisms, including ceramide synthesis [19]. However, a systemic study of the selectivity for the target cells (CSCs vs. BCCs) was not reported, and the molecular mechanism in colon CSCs has not yet been clearly elucidated. We and others suggested that the SCD1 activity may be required for Wnt signaling because MUFAs generated by this enzyme are essential for the ligand modification.

Here, we showed that the pharmacological intervention may be used for the selective targeting of colon CSCs as a treatment for patients with colon cancer. In this study, CSCs derived from 4 colon adenocarcinoma cell lines showed more than 100-fold greater sensitivity to the known SCD1 inhibitors than BCCs. SCD1 inhibition exclusively induced apoptosis in CSCs. Ultimately, we demonstrated the CSC-specific inhibitory effects are primarily mediated by alterations in Wnt and NOTCH signaling.

## 2. Materials and Methods 

### 2.1. Cells and Materials

Human colon cancer cell lines (HT29, SW480, HCT116, and HCT15) were purchased from the American Type Culture Collection (ATCC, Manassas, VA, USA) or Korean Cell Line Bank (Seoul, Korea). They were maintained as bulk culture cells (BCC) in Dulbecco’s modified eagle’s medium (DMEM, Invitrogen, Waltham, MA, USA) or RPMI 1640 (Invitrogen) supplemented with FBS (Fetal bovine serum, Invitrogen). For cancer stem cell (CSC) enrichment, they were cultured in DMEM/F12 containing B27 supplement and 20 ng/mL EGF (all from Invitrogen) in a polyHEMA (Sigma–Aldrich, St. Louis, MO, USA)-coated dish as described previously [20]. All cultured cells were maintained at 37 °C in a humidified atmosphere of 5% CO_2_. Plurisin #1 and MF-438 were obtained from Selleckchem (Houston, TX, USA) and Merk Millipore (Burlington, MA, USA), respectively.

### 2.2. Cell Proliferation Assay and CSC Sphere Proliferation Assay

The cells were reverse transfected with siSCD1, and siControl (NC) obtained from IDT (Coralville, IA, USA) using Lipofectamine^®^ RNAiMAX (Thermo, Waltham, MA, USA) in accordance with the manufacturer’s protocol. Cell viability was evaluated as previously published [20]. Four thousand cells were seeded in every well of a 96-well plate. After 24 h, they were treated with a different concentration of SCD1 inhibitors. After 2 days, MTT (3-(4, 5-Dimethylthiazol-2-yl) 2,5-diphenyltetrazolium bromide, Sigma–Aldrich) was added to each well with 250 µg/mL for 2 h at 37 °C. Supernatant was completely removed and 100 µL of DMSO was added to each well to dissolve the formazan crystals. The optical density was measured at a wavelength of 570 nm on an ELISA reader (Bio-Tek, Winooski, VT, USA) and the relative cell density was calculated.

### 2.3. Electron Microscopy, Cell Cycle Assay and Apoptosis Assay

Cells were fixed with PBS-based 4% paraformaldehyde for 20 min, treated with ethanol, and dehydrated for 5 min. After being treated with hexamethyldisilazane (Sigma–Aldrich, St. Louis, MO, USA), they were coated with gold and observed by a scanning electron microscope, JSM-7600F (Jeol, Akishima, Japan). For cell cycle analysis, the ethanol-fixed, RNAse (10 mg/mL, Sigma–Aldrich)-treated cells were stained PI (propidium iodide, 1 mg/mL, Sigma–Aldrich). For apoptosis analysis, cultured cells were stained with PI and and FITC-labeled Annexin V (BD, Franklin Lakes, NJ, USA). The cells were analyzed with Caliber (BD) and the data were further analyzed with Flowjo software (V10, BD). The statistical significance was measured by Student’s *t*-test. 

### 2.4. Western Blotting

The cell lysates were prepared as described previously [20]. Briefly, the Tris-based buffer containing phosphatase inhibitors and protease inhibitors (Roche, Basel, Switzerland) was applied and cleared with centrifugation. The protein in soluble fractions was quantified using a BCA kit (Thermo) and standard western blotting was performed using PVDF membrane (Merk Millipore, Boston, MA, USA). The antibodies used were as follows: α-tubulin (sc-23948) and PARP1/2 (sc-7150) were purchased from Santa Cruz (Santa Cruz, CA, USA). SCD1 (ab19862) was purchased from Abcam (Cambridge, UK). Cleaved active caspase-3 (#9661) was purchased from Cell Signaling Technology (Danvers, MA, USA). The secondary antibodies conjugated with horseradish peroxidase were purchased from Cell Signaling Technology. The signals were visualized using SuperSignal^®^ Femto (Thermo) and X-ray films (Agfa, Mortsel, Belgium).

### 2.5. Limited Dilution Assay and Mouse Experiment

The bulk cultured cells (BCCs) were treated SCD1 inhibitors. After 24 h, they were placed in 96-well polyHEMA-coated plates at various seeding densities (1~128 cells/well) in the CSC culture medium previously described. After 5 days, the fraction of wells not containing CSC spheres for each plating density was calculated and plotted against the number of cells per well.

All mouse experiments were pre-approved by IACUC of Sookmyung Women’s University. Well-proliferating HT29 cells (500,000) in 50 μL of phosphate buffered saline were injected into tail veins of 6-week-old male BALB/c Nude (nu/nu) mice (Koatech, Korea) mice. MF-438 was suspended in 0.6% carboxymethyl-cellulose (Sigma Aldrich) and orally administrated (10 mg/kg) daily for 13 days, and survival was examined until all the control group mice died. For the control, 5 mice were used and for the MF-438 treatment, 6 mice were used. The survival curve was plotted and the statistical significance of the difference was calculated with a log rank test using Prism (Graphpad, San Diego, CA, USA).

### 2.6. Quantitative RT-PCR

Total RNA of BCCs and CSCs was extracted with TRIsure™ (Bioline, London, UK). The CSCs spheres treated with MF-438 or vehicle (DMSO) for 24 or 48 h were collected, and the RNA was extracted. RNA was reverse-transcribed into cDNA with the SuperScriptIIFirst Strand cDNA Synthesis kit (Invitrogen) using random hexamers as primers. The quantitative RT-PCR was performed using the primers previously published [20] using Step One Plus (ABI, Foster City, CA, USA) and a PrimeTime^®^ Gene Expression Master Mix from MBiotech (Gyeongki, Korea). The expression level of each mRNA was measured and normalized to the expression level of L32 or β-actin and the relative expression was calculated based on the 2^−ΔΔCT^ method.

### 2.7. Bioinformatics Analysis of Clinical Data

TCGA PanCancer Atlas data set was downloaded from cBioPortal (www.cbioportal.org). In this analysis, 593 patients were analyzed and mRNA expression z-scores were used which were compared to the expression distribution of all samples. Box and whisker plots were plotted using GraphPad Prism. The significance of difference between mRNA levels of two groups (25% upper and lower) was examined statistically by unpaired Students’ *t*-test. 

## 3. Results

**Figure 1:**
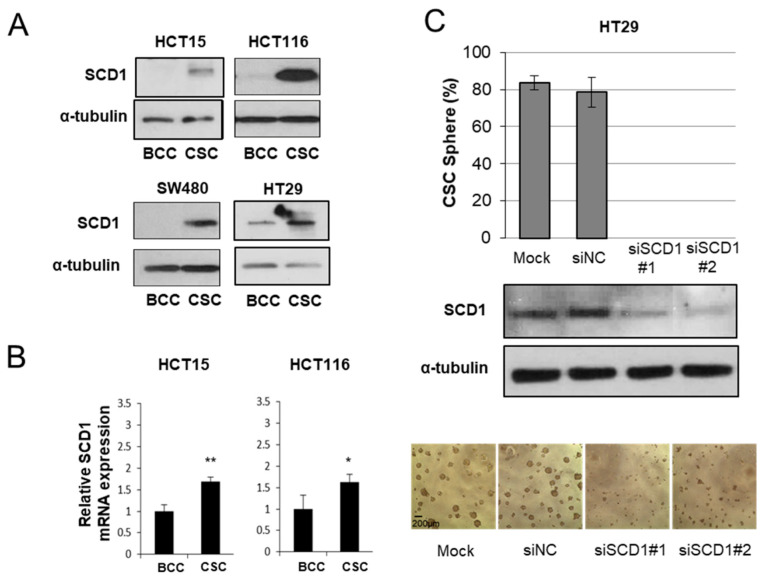
Cancer stem cells (CSC) increase the expression of SCD1, which is essential for CSC survival. (**A**) CSC expressed the SCD1 protein more than bulk cultured cells (BCC). (**B**) Enhanced expression of SCD1 transcripts in CSC than that of BCC (quadruplicates). *, *p* < 0.05; **, *p* < 0.01 in Student’s *t*-test. (**C**) SCD1 is essential for CSC maintenance. Two SCD1 siRNA (siSCD#1 and siSCD#2, from IDT) and siNC (negative control, scrambled, from IDT) were reverse transfected and the CSC sphere were counted after 5 days (6 replicates). The scale bar represents 200 micrometers.

We first tested if the colon CSCs expressed the SCD1 enzyme at higher levels by analyzing 4 colon adenocarcinoma cell lines. The BCCs were cultured in attached conditions with serum, as most carcinoma cells are cultured, and CSCs were enriched as described earlier [21]. When the cells were in the logarithmic phase of growth, the cells were harvested and the expression of SCD1 was compared. The SCD1 protein (Figure 1A) and mRNA (Figure 1B) were expressed at much higher levels in all the tested colon adenocarcinoma cell lines under CSC-enriched culture conditions. We then tested whether the expression of these genes was essential for CSC proliferation using siRNA transfection. Although the transfection reagents alone slightly inhibited CSC sphere formation, 2 siRNAs targeting SCD1 completely abolished CSC sphere formation after 5 days in culture, suggesting the absolute requirement for the SCD1 enzyme (Figure 1C). The decrease of SCD1 by siRNAs did not diminish the cell numbers in BCC cultures (Supplementary Materials Figure S1).

**Figure 2:**
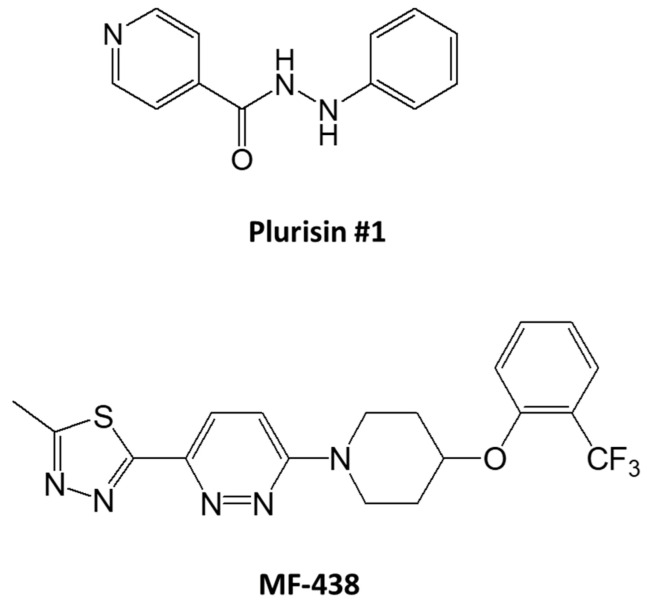
Structure of SCD1 inhibitors, Plurisin #1 and MF-438.

This molecular biological targeting of SCD1 prompted the hypothesis that the fatty acid denaturation activity is a vulnerable target for CSC elimination. We took advantage of two independently developed and structurally unrelated pharmacological inhibitors of the enzymes, Plurisin #1 [22] and MF-438 [23], to test this hypothesis (Figure 2).

**Figure 3:**
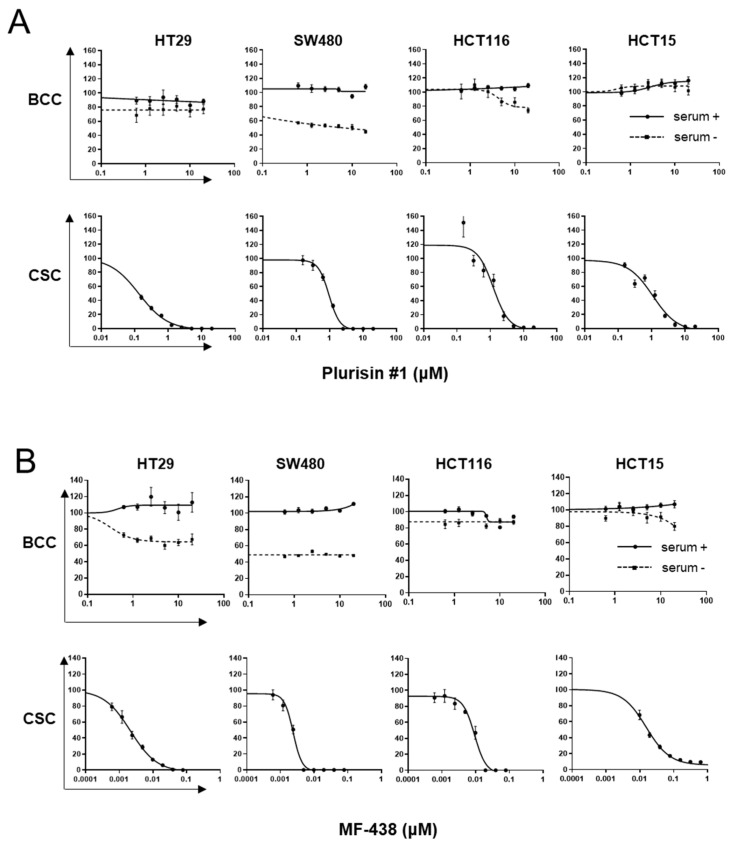
SCD1 inhibitors suppress colon CSCs but not the BCCs. Four colon cancer cell lines, cultured as BCC (with or without serum, each 6 replicates) and cultured for CSC enrichment were treated with the SCD1 inhibitors (each 6 replicates). (**A**) Plurisin #1, (**B**) MF-438, and the relative viability and number of spheres (bigger than 100 μM in diameter) were counted.

The treatment with Plurisin #1 was not toxic to the proliferating cells in all tested cell lines cultured under BCC conditions. However, the number of CSC spheres substantially decreased after treatment with the low concentration of Plurisin #1. The effects of MF-438 were more prominent; a micromolar treatment with this inhibitor only suppressed proliferation to a limited extent, regardless of the presence of serum under BCC culture conditions. However, the CSC enrichment was well suppressed by 10 nM MF-438, showing at least 100-fold greater sensitivity under this condition (Figure 3B). The effect on selective CSC elimination was observed in all 4 cell lines tested.

**Figure 4:**
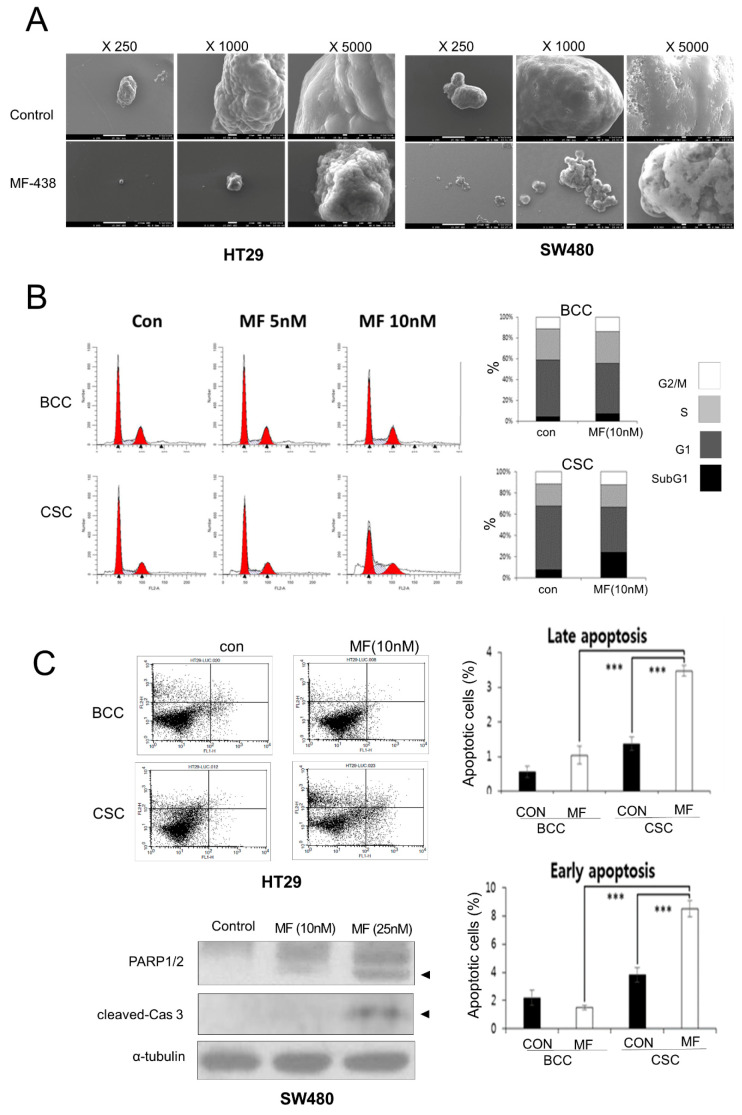
SCD1 inhibition led apoptosis of CSCs. (cancer stem cells). (**A**) Scanning electron microscope images demonstrate apoptotic cells in MF-438 (MF) treated CSCs. (**B**) SCD1 inhibited HT29 CSCs died at G1 (*n* = 3 for each) but the bulk cultured cells (BCC) did not. (**C**) SCD1 inhibition-led cell death was through apoptosis; (*n* = 3 each); Y-axis, PI staining; X-axis, Annexin V staining. Cells were cultured as BCC and CSC, with or without MF-438, for 72 h. Arrowheads mark the cleaved PARP1/2 and cleaved Caspase 3 (C-Cas3). Representative images of at least 2 independent experiments shown. ***, *p* < 0.001 in Student’s *t*-test.

We observed the cell membrane morphology using scanning electron microscopy to more carefully examine the mode of CSC elimination. MF-438 treated CSCs showed a typical apoptotic granular morphology (Figure 4A), consistent with a previous report [24]. The BCCs did not display this morphological change by the inhibitors (Supplementary Materials Figure S2). No specific cell cycle changes or dying cells were identified in BCCs treated with SCD1 inhibitors. However, the blockade of the SCD1 enzyme decreased the percentage of CSCs in G1 phase and increased the percentage of dead CSCs (Figure 4B). Annexin V and PI staining showed a greater increase in the population of apoptotic CSCs treated with MF-438. Analyses of the levels of cleaved poly (ADP-ribose) polymerase (PARP) and cleaved caspase 3 also showed that the CSCs undergo apoptosis after treatment with MF-438 (Figure 4C).

**Figure 5:**
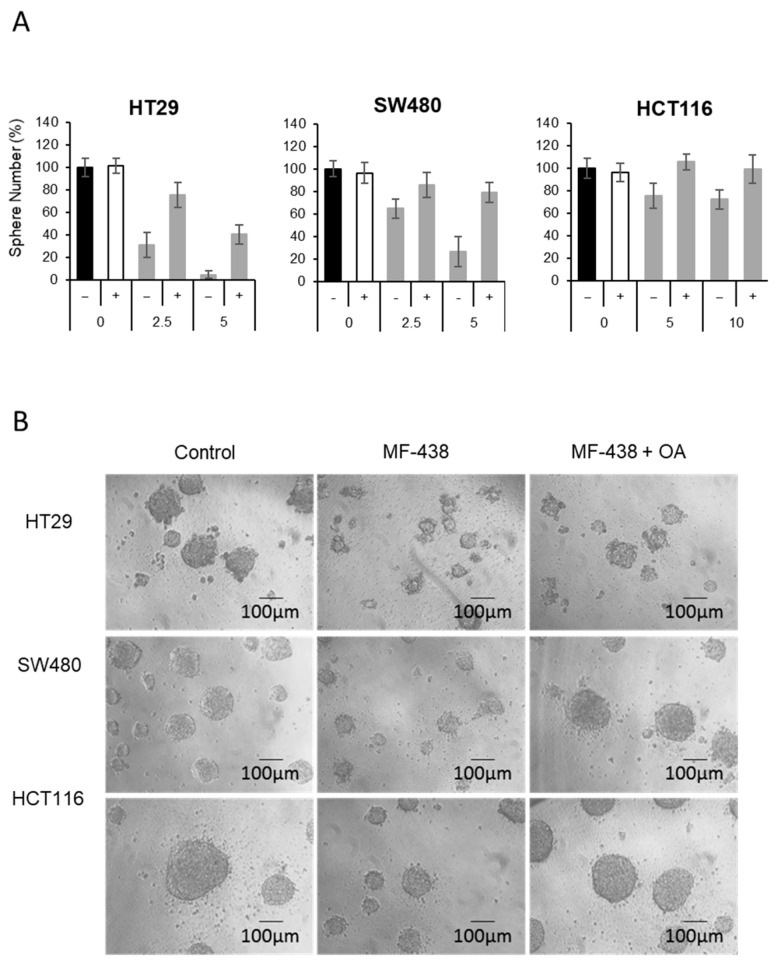
SCD1-generated monounsaturated fatty acid is necessary for CSC survival. (**A**) The SCD1 inhibitor was treated with or without oleic acid (OA; 18:1, monounsaturated, 200 μM) and the number of CSCs spheres bigger than 100 μm in diameter was counted after 5 days. Results of 6 replicates. (**B**) Representative images of CSC spheres in (**A**) under microscope (scale bars represent 100 μm).

Although we tested two independent SCD1 inhibitors and siRNA transfection to assess the CSC-selective anti-proliferation activity, we questioned if the enzymatic activity of SCD1 for fatty acid desaturation is essential for CSC survival or whether other unknown substrates and products are related to this process. Since the main product of SCD1 is palmitoleic acid (16:1) and oleic acid (18:1), we treated oleic acid with or without SCD1 inhibitors and found that those treatments nicely rescued the suppressive effects of the inhibitors on all three tested CSC lines (Figure 5A,B). 

**Figure 6:**
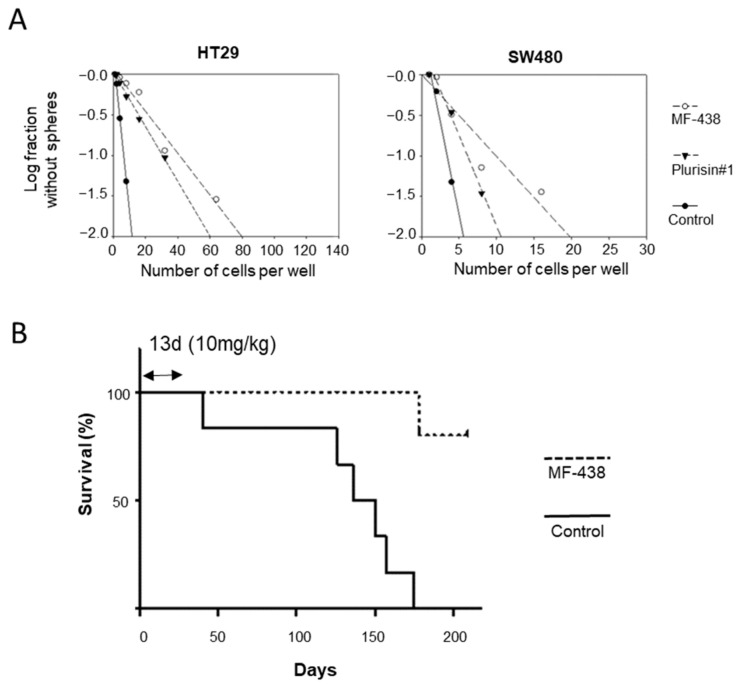
The CSC potential required SCD1 activity in vitro and in vivo. (**A**) Self-renewal activity of colon cancer cell was decreased by SCD1 inhibition. The cells cultured as BCC were treated with SCD1 inhibitors and subjected to limiting dilution assay. After 5 days, the CSC sphere negative wells increased by the inhibitors treatment. (**B**) The oral treatment of SCD1 inhibitor (10 mg/kg per day, for 13 days) prolonged survival of HT29-tail vein injected nude mice (*n* = 6 for control, *n* = 5 for MF-438). The obtained *p*-value between two groups using logrank tests was 0.0014.

We then tested if the CSC-selective inhibitory effect of SCD1 inhibitors indeed targeted the CSC population by measuring the most essential CSC characteristics, namely, the self-renewal property, in vitro and in vivo (Figure 6). The proliferation of BCCs was not affected by SCD1 inhibition, based on the results of the MTT assay. However, the BCCs contain a small number of CSCs. Therefore, we hypothesized that the small number of CSCs may be eliminated by the MF-438 treatment. BCCs were treated with 10 nM and 1 μM of MF-438 and Plurisin #1, respectively, for 24 h and subjected to the limited dilution assay, a widely used analysis for CSCs in vitro. The self-renewal of CSCs was clearly reduced in the BCC population pretreated with the SCD1 inhibitors (Figure 6A). The in vivo effect of SCD1 inhibition on CSCs was also tested. Because the CSCs are believed to be responsible more in in vivo self-renewal for tumor initiation than for tumor volume increase [25], the tail vein injection for metastasis may be more relevant for in vivo CSC potential test [2]. The administration of MF-438 to mice that received a tail vein injection of HT-29 CSCs significantly improved the survival, indicating that the anti-CSC potential was also maintained in vivo.

**Figure 7:**
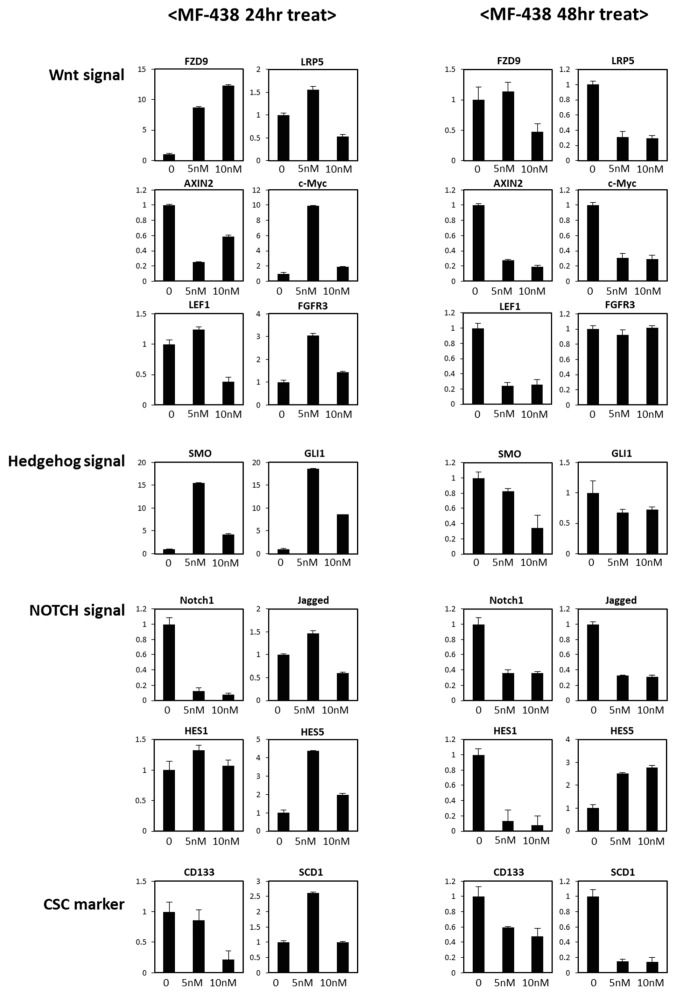
Time-dependent decrease of key stem cell molecules in CSC (cancer stem cells) by SCD1 inhibitor. Quantitative RT-PCR of the Wnt, Hedgehog, Notch signal key molecules mRNA and CSC marker mRNA after 24 and 48 h treatment of MF-438 (5 and 10 nM). Axin2 and Notch1 expression decrease were detected in advance of many key molecules decrease. The key molecules for each signaling are also the target genes of each signaling (Wnt–Axin2 and Lef-1; Notch–Notch1 and Hes1; Gli–Gli1).

We then investigated what signaling pathways were primarily blocked by the SCD1 inhibitors and induced CSC death. We measured the expression of 14 CSC-related genes in SW480 CSCs after treatment with 5 and 10 nM MF-438 for 24 and 48 h (Figure 7). At 48 h, most of the genes involved in the Wnt-, Notch-, and SHH-signaling pathways were expressed at significantly lower levels in the CSCs cultured with 5 or 10 nM MF-438. These genes included LRP5, Axin2, LEF-1, and c-MYC in the Wnt-signaling pathway, Smo and Gli1 in the SHH-signaling pathway, and Notch1, Jagged, and Hes1 in the Notch-signaling pathways. All 3 major signaling pathways that are most important for stem cells and CSCs appeared to be suppressed drastically. However, CD133 expression and SCD1 expression were decreased in these cells, suggesting that the cells with CSC potential may have already died at this time point that resulted in the downregulation of most CSC signaling genes.

At 24 h after treatment, when CD133 expression had not yet changed significantly and SCD1 expression was increased to respond to treatment with 5 nM SCD1 inhibitor, only the expression of Axin2 and Notch1 was suppressed. The 10 nM treatment may have eliminated the CSCs, as evidenced by CD133 expression and the feedback cell elimination, allowing SCD1 expression to return to the control level. Notably, LEF-1 expression also decreased after 24 h of treatment with 10 nM MF-438, preceding the decrease in the expression of many other Wnt target genes. Collectively, although the expression of most downstream genes in the 3 major stem cell signaling pathways decreased at 48 h, we only observed decreased expression of Wnt and Notch signaling target genes in cells treated with the low concentration at the earliest time point. The temporal increase in the expression of most other signaling genes may represent a negative feedback response to that suppression.

**Figure 8:**
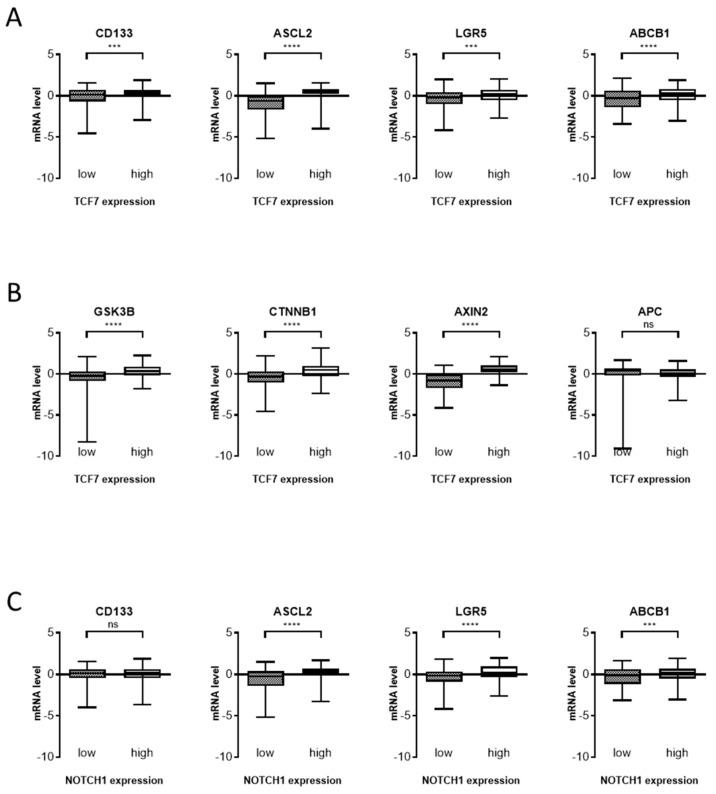
Expression of Notch- and Wnt-signaling genes are associated with colorectal stem cell markers. Box and whisker plots showing each gene’s mRNA levels in two groups. (**A**) Stem cell marker expression and TCF7 expression. (**B**) TCF 7 gene expression and other Wnt signaling components’ expression. (**C**) Notch1 expression and stem cell marker expression. The patients were classified into “high” and “low” groups depending on TCF7 or Notch1 mRNA level (25% upper and lower, respectively). The TCGA PanCancer Atlas database included 593 patient and mRNA expression z-scores relative to all samples. *p* > 0.05 was considered not significant (ns); ***, *p* < 0.001; ****, *p* < 0.0001.

Since the RT-PCR test strongly suggest Wnt and Notch signaling are primarily suppressed by SCD1 inhibition, we tested if these signaling genes’ expression has correlation with the CSC markers’ gene expression (Figure 8). Several colon CSC marker genes, CD133, ASCL2, LGR5, and ABCB1, were highly expressed in the TCF7 gene high specimen (Figure 8A). TCF7 is a central player in Wnt signaling in the colon and showed good correlation with other Wnt-signaling molecules, GSK3b, CTNNB1, and Axin2, except APC (Figure 8B). It may be due to the fact that APC loss of function mutation is predominant in colorectal cancers. Notch1 expression also showed associated expression of the colon CSC markers, ASCL2, LGR5, and ABCB1(Figure 8C). These data strongly suggest that Wnt and Notch signaling do pivotal roles in colon CSCs.

## 4. Discussion

In the last half century, many efforts in cancer research have revealed the characteristics of cancers. However, this fearful malady is still a leading cause of death worldwide, mainly due to metastasis and recurrence. Recent advances in immunotherapy and targeted therapy may provide insights into methods to improve the prognosis of patients with cancer. The concept of a CSC targeting strategy is also attracting more interest due to its expected role in acquiring resistance. In the effort to identify a CSC-specific target that will not damage other normal cells, we and other researchers recently identified a lipid desaturase, SCD1, as a novel target for CSC targeting. In the present study, the SCD1 targeting strategy suppressed the two most pivotal signaling pathways in CSCs, Notch and Wnt, leading to CSC-specific apoptosis in colon cancer.

Wnt and Notch signals are the most crucial signaling for the activity of epithelial stem cells [26]. Of the two, many mutations in Wnt signaling, including loss of function of APC and gain of function β-catenin which result in sustained Wnt-signaling activation, are found in colorectal cancers [27]. Though the mutations are not frequently found in Notch signaling, they also play pivotal roles in the intestinal stem cells and cancer stem cells [28,29]. 

Recently, we identified substantial differences in the lipidome profile between CSCs and BCCs [11,18]. Surprisingly, the composition of monounsaturated fatty acids (MUFAs) in CSCs was much greater than in BCCs of glioblastoma, suggesting a role for the MUFA-generating enzyme SCD1 in CSCs [17]. An independent study of ovarian cancers also showed an increased composition of MUFAs and ovarian CSCs [30]. We also observed an increase in the MUFA composition in colon CSCs than in BCCs [11]. The main MUFAs generated by SCD1 in human cells are palmitoleic acid or oleic acid and these can be components of many lipid molecules. Yet, the most well-known signaling modulated by MUFA is Wnt signaling because the Wnt ligand must be tagged with this palmitoleic acid by an enzyme porcupine and this is essential for the Wnt ligand secretion. 

Interestingly, the pharmacological SCD1 inhibition results in oleic acid (18:1) depletion and changes in sphingomyelin (SM d18:1/20:0 or d16:1/22:0) and phosphatidylcholine (PC; p-18:0/18:1)) levels [11]. These two phospholipids and cholesterol are major components that form lipid rafts, which mediate many cell signaling events [31,32,33]. Lipid rafts are subdomains of the membrane with distinct protein and lipid compositions that are involved in signal transduction across the plasma membrane [34]. LRP6 and NOTCH proteins mediate Wnt and Notch signaling, respectively, in lipid rafts [35,36]. These signals may be the most important signaling pathways for CSC proliferation and stemness maintenance [6,37,38]. Therefore, the MUFA-generating enzyme may be required to ensure the proper composition of CSC lipid rafts, and the blockade of SCD1 and subsequent inhibition of Wnt and Notch signaling may be due to the changes in SM (d18:1/20:0 or d16:1/22:0) and PC (p-18:0/18:1). These results suggest that, in addition to the more direct inhibition of Wnt ligand palmitoleic acid modification [39,40], changes in other lipid metabolites resulting from SCD1 inhibition may also lead to Notch and Wnt signaling blocking and induce CSC apoptosis. 

Although we tested many genes related to these signaling pathways, the gene expression analyses may not reveal the direct target of SCD1 inhibition. Changes in the transcripts may represent the outcome of changes in upstream signaling pathways. Of the Wnt target genes displaying altered expression, the decrease in Axin2 expression preceded the changes in the expression of all the other genes in cells treated with the 5 nM inhibitor for 24 h, a time point before the expression of a CSC marker, CD133, decreased. Axin2 is a main component and primary target of Wnt signaling. Wnt activates β-catenin and induces the formation of a complex with SP1 that binds the promoter of the Axin2 gene to activate the transcription in colon cancer [41], and Axin2 expression is quickly suppressed following dephosphorylation of LRP6 in the lipid raft by a porcupine inhibitor [40]. The downregulation of all other Wnt downstream genes was observed later at 48 h or after treatment with a higher concentration. Therefore, we conclude that Wnt signaling is blocked by SCD1 inhibition. However, there are more genes (including GSK3β, APC, β-catenin, and CK1) positively and negatively contributing to the Wnt pathway. In addition, the non-canonical Wnt pathway also contributes in CSCs [42]. Further studies regarding these genes’ expressions in protein and mRNA levels may give us further understanding as to how Wnt signaling is modulated by SCD1 in CSCs.

Colon stem cells employ a positive feedback mechanism through the direct binding of the Notch1 intra cellular domain to its own promoter [43]. The strong and prompt suppression of Notch1 transcription at 24 h may be explained by this feedback from the suppressed Notch signaling. Jagged and Hes1 downregulation were observed at 48 h, but Hes5 is frequently upregulated to respond to a blockade of Notch signaling [44]. The increase in SCD1 expression in cells treated with 5 nM inhibitor for 24 h was interesting because it may suggest that the inhibition of SCD1 enzymatic activity caused the CSCs to increase SCD1 gene expression. The loss of SCD1 expression, similar to CD133, at 48 h may show the value of SCD1 as a noble CSC marker. At 48 h, the decreased expression of most of the CSC signaling genes and the CSC markers may indicate that the apoptotic cells were in the CSC population under this condition.

Since the Wnt pathway is generally activated in colon cancer cell lines due to APC mutation, the 100-fold or greater difference in sensitivity to MF-438 between CSCs and BCCs is interesting [45]. Indeed, most colon cancer cells are heterozygous for APC mutations. In addition, those mutations may blunt the ligand (Wnt) dependence of the cells and desensitize them to Wnt inhibition. This question may have a few possible explanations. First, the cells with a partial loss of function mutation in APC gene may still depend on the Wnt ligand signal that may be more critical in the CSC context in which Wnt plays more pivotal roles. Second, the Notch signal may perform critical functions in CSCs, but not in BCCs. Third, these two pivotal signaling pathways for CSC self-renewal interact [37] in a synergistic manner. Indeed, Notch directly interacts with β-catenin [46,47] to enhance the transcription potential [48]. Therefore, the dual targeting leads to an enhanced and synergistic effect on CSCs only, but not BCCs, where Notch signaling may play less important roles.

The clinical data presented also showed the importance of Wnt and Notch signaling in colon CSCs. With our previous report [11] that the Wnt and Notch signaling gene expression is elevated in SCD1 high specimen of colon tumors; these data strongly support the reciprocal regulatory network among these markers and signaling molecules. 

Further in-depth animal studies are also warranted to examine the potential use of SCD1 inhibitors in anti-CSC therapy. Considering that CSCs are increased by conventional chemotherapy [25], the combined treatment of SCD1 inhibitors with a chemotherapeutic agent may be appropriate. 

## 5. Conclusions

Collectively, here we showed that SCD1 inhibition led selective CSC elimination by suppressing Notch and Wnt signaling. This dual targeting effect may be caused by changes in lipid metabolites, such as MUFAs and SM, and the synergistic interaction of both signaling pathways (illustrated in the Graphical Abstract). Currently, several SCD1 inhibitors have been developed and are used in the clinic in a non-oncology local application setting. However, their systemic application has not yet been approved. Further in-depth studies are warranted for a clinical evaluation of the SCD1 inhibitors as CSC-targeting agents to improve efficacy of other conventional therapeutics. 

## Data Availability

All data are included in the paper and supplementary materials except the clinical data which is available from cBioportal portal (https://www.cbioportal.org/).

## Supplementary Materials

The following are available online at https://www.mdpi.com/2073-4409/10/1/106/s1, Figures S1: SCD1 inhibition did not reduce BCC numbers. Figure S2: Scanning electron microscope images of non-apoptotic cells in BCC after MF-438 treatment. Figure S3: Original image of the Western blots.
