# Supplementary material for: Targeting a Lipid Desaturation Enzyme, SCD1, Selectively Eliminates Colon Cancer Stem Cells through the Suppression of Wnt and NOTCH Signaling
Source: Cells. 2021 Jan 8;10(1):106. doi: 10.3390/cells10010106 (PMC7826607; doi:10.3390/cells10010106)

Supplementary Materials Figure S1. Decresae of SCD1 did not reduce cell number cultured in bulk condition. siRNA reverse-transfected HT29 BCC (Bulk culltured cells) are plated and counted aftre 3 days. Mock is without siRNA. siNC is scramled siRNA for sontrol and siSCD1#1 and scSCD1#2 are siRNAs against SCD1 but with differernt sequences. All from IDT.

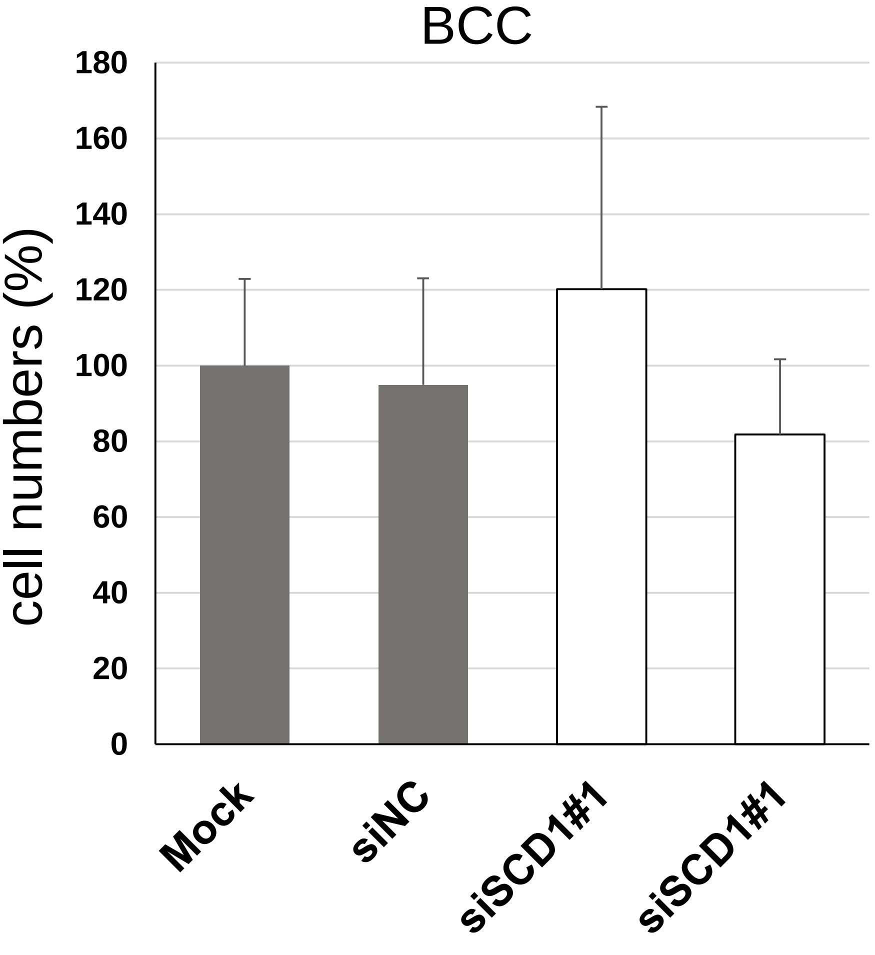

Supplementary Materials Figure S2. SCD1 inhibition did not induce apoptosis in BCC (bulk cultured cells). Scanning electron microscope images demonstrate no apoptotic morphologies in BCC by MF-438 or DMSO (control).

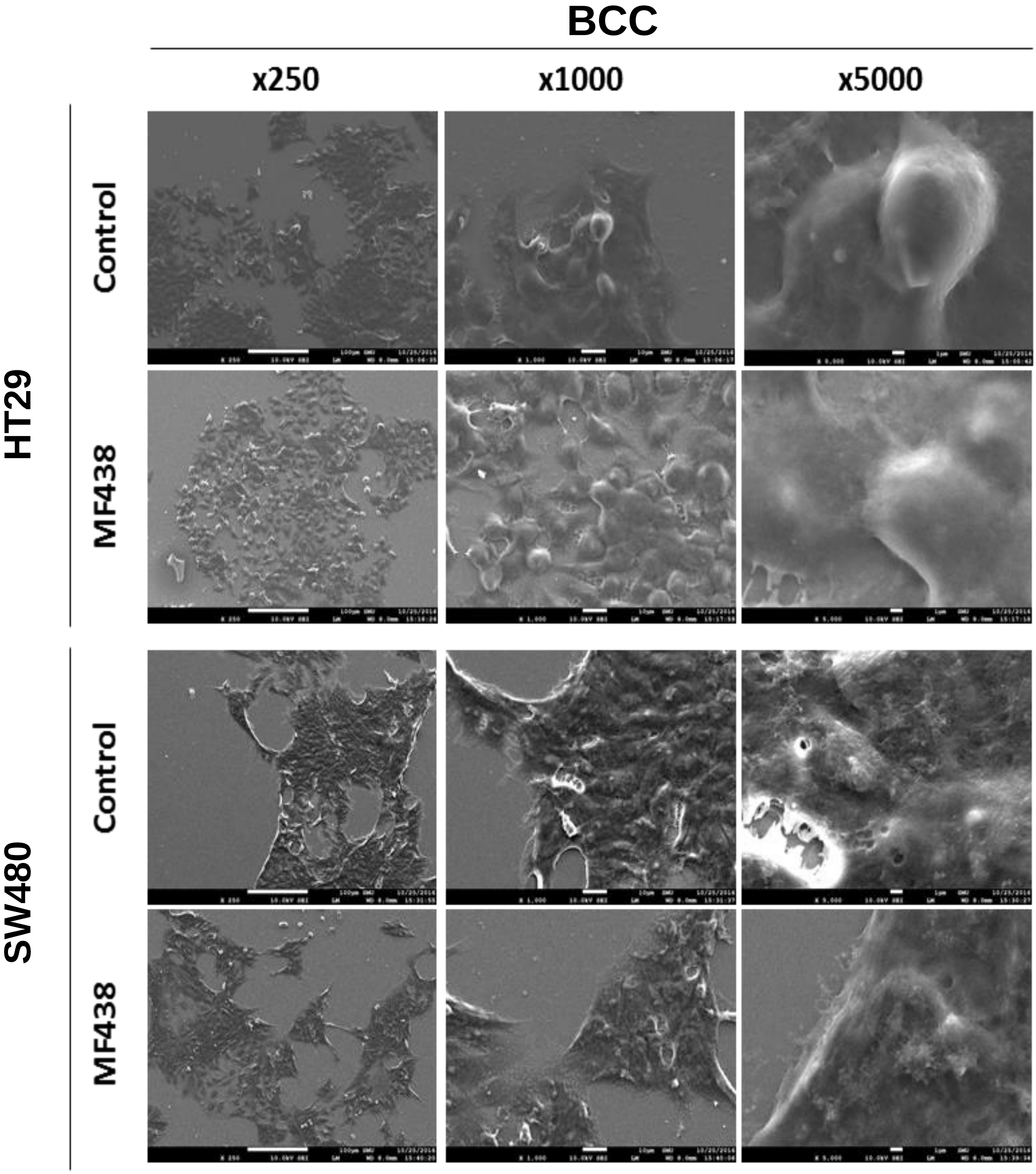

Supplementary Materials Figure S3. Original image of the Western blots used.

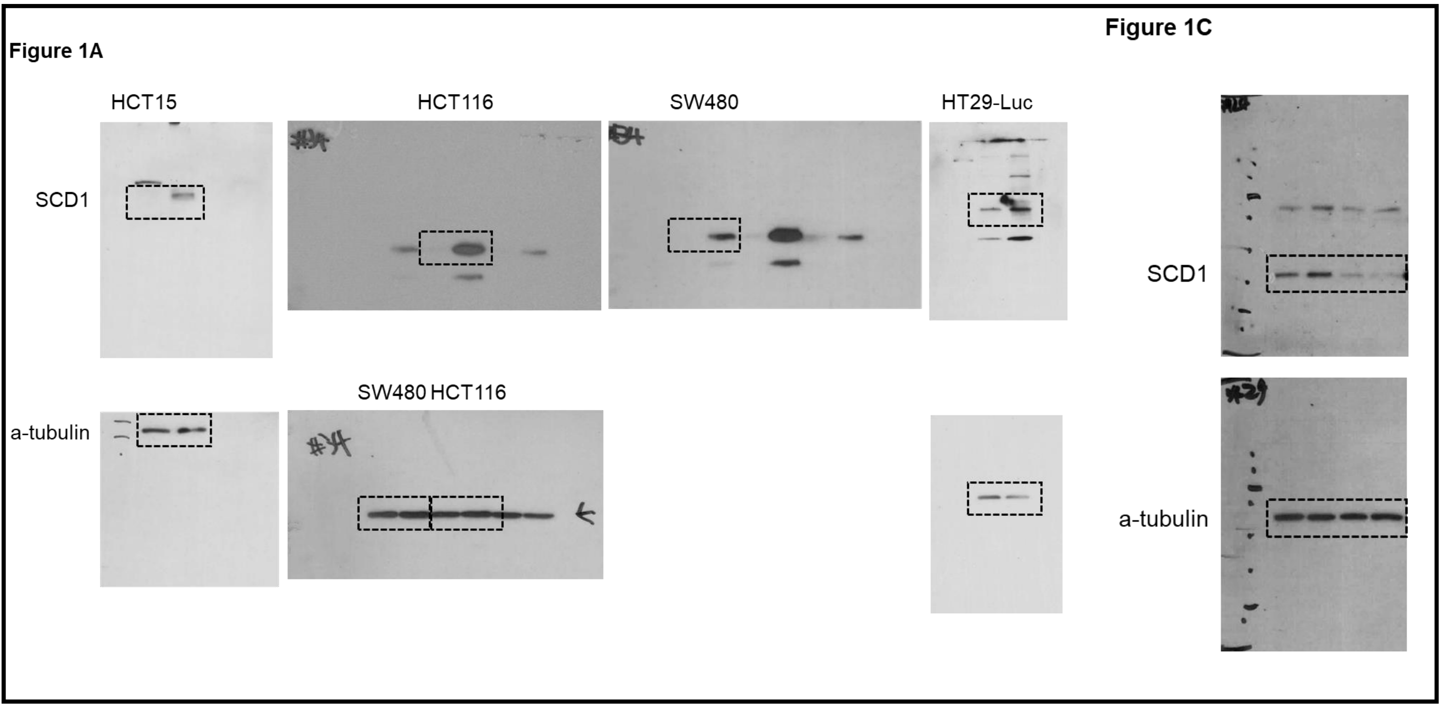

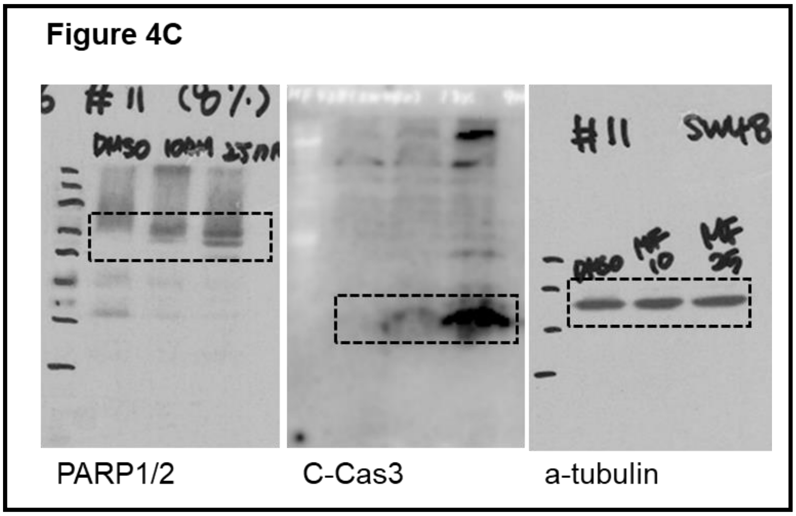

Supplement: Supplementary file 1 [file cells-10-00106-s001.pdf]
